# HEALTHY BRAIN AGING: OPPORTUNITIES AND CHALLENGES

**DOI:** 10.1093/geroni/igae098.1183

**Published:** 2024-12-31

**Authors:** Andrew Sixsmith

**Affiliations:** Chair

## Abstract

Dementia and cognitive decline are amongst the greatest challenges of modern times and have a major impact on individuals, families, and society. Although there are currently no effective treatments or cures for dementia-related conditions, there is huge scope for preventative measures to improve brain health as people grow older. By developing and implementing real-world solutions that address known modifiable risk-factors, the decline of cognitive health and the onset of dementia may be delayed or prevented. With the right combination of policies, services and changes in health-related behaviours, it may be possible to expand the healthspan of people, where most people can live healthier, active lives as they grow older. Our interdisciplinary symposium comprises four papers that examine the challenges and opportunities for brain health and healthy aging. Sylvain Moreno a discusses heterogeneity in neurocognitive aging, challenging the predominantly linear view of cognitive decline with age. Holland and colleagues draw on a Delphi consensus study to explore opportunities for developing Interventions for cognitive frailty, the potential to delay, prevent or reverse cognitive frailty, and how people with lived experience can contribute to clinical and research expertise, White, Sixsmith and Fang explore the role of community-based organisations in supporting healthy aging, focusing on practical strategies for mobilizing community assets and resources to promote healthy aging. Boot, Dilanchian and Kalantari examine the role of technologies to support healthy brain aging, particularly the potential of extended reality technologies to support older people with and without cognitive impairments.

